# Decreased Control and Enhanced Reactivity: Dual Dysregulation Pattern of Cross‐Frequency Coupling in Emotional Susceptibility of Non‐Clinical Insomnia

**DOI:** 10.1002/cns.71031

**Published:** 2026-07-29

**Authors:** Siyu Li, Qingwei Chen, Wenxin Liu, Lingling Zhang, Zhiguo Jiang, Taotao Ru, Guofu Zhou

**Affiliations:** ^1^ Guangdong Provincial Key Laboratory of Optical Information Materials and Technology, Institute of Electronic Paper Displays, South China Academy of Advanced Optoelectronics South China Normal University Guangzhou China; ^2^ Provincial Key Laboratory of Multimodal Perceiving and Intelligent Systems, College of Information Science and Engineering Jiaxing University Jiaxing China; ^3^ Lab of Light and Physio‐Psychological Health, National Center for International Research on Green Optoelectronics South China Normal University Guangzhou China; ^4^ School of Psychology South China Normal University Guangzhou China

**Keywords:** cross‐frequency coupling, emotional susceptibility, insomnia disorder, negative bias, phase‐amplitude coupling

## Abstract

**Objective:**

Insomnia involves impaired emotional attention, yet the underlying neurophysiological mechanisms remain unclear. This study investigated neurobehavioral characteristics of emotional processing in insomnia, specifically examining modulations by emotional valence and intensity.

**Methods:**

Twenty‐five individuals with non‐clinical insomnia disorder (NCID) and 22 normal controls (NC) performed a two‐choice oddball task with emotional images (varying in valence and intensity) during task‐state EEG recording. Phase‐amplitude coupling (PAC) and time‐frequency analyses were utilized to assess neural dynamics.

**Results:**

The NCID group responded averagely slower and showed distinct negative bias, with response being faster to negative versus positive affective images. PAC analysis confirmed a dual dysregulation: the NCID group showed significantly reduced delta‐beta coupling in frontal regions and significantly increased theta‐beta coupling in occipital regions. Importantly, these coupling alterations were intensity‐dependent; the NCID group showed a blunted dynamic range across intensities and lower frontal coupling specifically for high‐intensity negative stimuli. Time‐frequency analysis further revealed increased posterior theta power in the NCID group, while beta oscillations showed enhanced responses to negative stimuli in both groups.

**Conclusion:**

Insomnia is characterized by altered emotional susceptibility involving intensity‐dependent alterations in cross‐frequency coupling (CFC). These findings clarify the neurophysiological mechanisms of emotional dysfunction in insomnia and highlight CFC as a potential biomarker for emotional regulation impairments.

## Graphical abstract

Insomnia disorder involves a dual dysregulation pattern: reduced frontal delta‐beta coupling (impaired control) and enhanced occipital theta‐beta coupling (heightened reactivity). These mechanisms underlie emotional susceptibility and negative attentional bias.

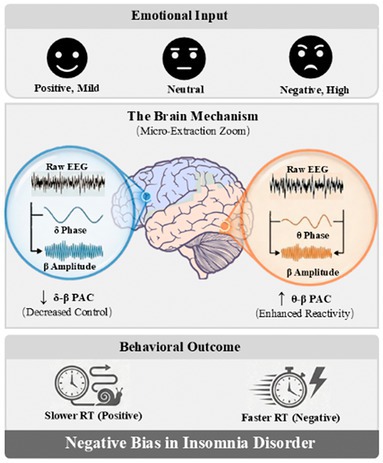

## 1 Introduction

Insomnia is a major public health issue in modern society [1]. It is characterized by difficulty initiating or maintaining sleep despite adequate opportunity [2]. A recent meta‐analysis indicated that approximately 12.4% of the adult population experiences insomnia symptoms [3]. Poor sleep quality severely impairs daily functioning, induces persistent fatigue, triggers emotional instability, and gives rise to notable impairments in attention and memory [4, 5, 6].

Longitudinal research utilizing cross‐lagged designs has documented a bidirectional relationship between sleep disturbances and affective disorders. Persistent insomnia increases the risk of depression and anxiety, while emotional dysregulation, in turn, worsens sleep problems [7, 8]. Furthermore, empirical studies have reported that insomnia induced specific deficits in emotion regulation [9], selective attentional biases toward threat‐related cues [10], and impaired recognition of negative stimuli in emotional working memory [11]. Although behavioral assessments have established these alterations, the underlying neurophysiological mechanisms are still unclear.

Moreover, the existing studies have focused on emotional valence, largely overlooking the moderating role of emotional intensity. Previous neuroimaging studies revealed that varying levels of emotional intensity triggered distinct neural responses, particularly in the medial temporal lobe structures related to alertness [12, 13]. Kyle et al. [14] reported that individuals with chronic insomnia perceived facial expressions as less intense, suggesting altered emotional sensitivity. However, the neural dynamics driving this intensity‐dependent processing remain unexplored.

Electroencephalography (EEG) provides the necessary temporal resolution to investigate the neurophysiological dynamics of emotional processing in insomnia. While traditional stimulus‐locked components mainly reflect local neural activity, localized task‐evoked spectral power provides relevant information about rhythmic brain activity during emotional processing. According to the Hyperarousal Theory, insomnia is characterized by persistent cortical activation [15]. Consistent with this perspective, previous event‐related spectral perturbation (ERSP) analyses indicate that sleep deprivation reduces localized theta oscillations during empathy for pain tasks [16], and emotional conflict processing elicits enhanced theta activity [17]. Individuals with non‐clinical insomnia disorder (NCID) also exhibited higher posterior theta and alpha power during emotion perception [18].

However, the standard single‐band spectral analysis approach often fails to capture the interplay between regulatory slow‐waves and arousal‐related fast‐waves [19]. Cross‐frequency coupling (CFC) holds the potential to reveal how oscillations of different frequencies interact to integrate neural information [19, 20]. Investigating these cross‐frequency interactions is crucial for characterizing the specific oscillatory deficits that maintain sleep‐dependent emotional dysfunction [21]. Phase‐amplitude coupling (PAC), defined as the modulation of high‐frequency amplitude by the phase of low‐frequency oscillations, serves as a key metric for cortical–subcortical connectivity. Previous studies have shown that the frontal delta‐beta coupling reflects prefrontal inhibitory control over subcortical regulatory circuitry [22]. Recent evidence indicates that sleep loss compromises this regulatory capacity, manifesting as altered delta‐beta coupling and elevated state anxiety [23]. Similarly, theta‐beta coupling reflects functional coordination across cortical regions and varies as a function of motivational states [24]. In the context of sleep disturbances, abnormal beta and theta activities frequently indicate neurophysiological hyperarousal and impaired emotional regulation during resting and task states [25, 26]. Yet, it remains unknown to what extent insomnia affects these CFC neural activities and whether they are modulated by the valence and intensity of emotions.

Therefore, in addition to exploring the behavioral responses during implicit emotional processing, the current study also examines the time‐frequency characteristics of neural activity, particularly the PAC patterns involved in implicit processing of emotional stimuli varying in both valence and intensity in individuals with NCID.

Based on the hyperarousal model and prior electrophysiological evidence, three specific hypotheses were proposed. First, given that frontal delta‐beta coupling reflects regulatory control during emotional processing [24, 27], individuals with NCID might exhibit attenuated delta‐beta coupling in frontal regions. Second, as theta‐beta coupling is linked to vigilance and arousal states [28], enhanced posterior theta‐beta coupling would be observed in the NCID group. Third, emotional intensity and valence would moderate the coupling of delta‐beta and theta‐beta [12, 13]. To complement these hypothesis‐driven assessments, stimulus‐locked event‐related potentials (ERP) and localized time‐frequency power were investigated as exploratory analyses.

## 2 Methods

### 2.1 Experimental Design

A 2 (Group: NCID, normal control (NC)) × 2 (Valence: Positive, Negative) × 3 (Intensity: High, Mild, Neutral) mixed design was implemented. The study protocol received approval from the local university ethics committee, and written informed consent was obtained from all participants.

### 2.2 Participants

A priori power analysis using G*Power indicated that the minimum sample size was 38, assuming a medium expected effect size of 0.25, a significance level of α = 0.05 and 80% power. Totally, 47 participants were recruited: 25 individuals with NCID (5 males, 20 females; mean age = 21.76 ± 2.14 years) and 22 NC (8 males, 14 females; mean age = 20.54 ± 2.32 years). Participants in the NCID group were screened according to DSM‐5: defined by a Pittsburgh Sleep Quality Index (PSQI) score > 7 [29], an Insomnia Severity Index (ISI) score > 7 [30], and symptom duration exceeding 1 month. Participants in the NC group were required to have PSQI score < 5 and ISI score < 5. Exclusion criteria for both groups included Beck Depression Inventory‐II (BDI‐II) scores ≥ 14 [31], substance abuse, and a history of other neurological or psychiatric disorders.

### 2.3 Experimental Materials

Stimuli consisted of 180 deviant images selected from the Chinese Affective Picture System (CAPS) [32], divided into positive and negative blocks. Within each block, images were stratified into high, mild, and neutral intensity categories, with each containing 30 images. A neutral image (valence = 5.59, arousal = 4.40) served as the standard stimulus. For the positive block, the three categories of deviant pictures differed significantly in valence (*M*_high_ = 7.13 ± 0.11; *M*_mild_ = 6.45 ± 0.17; *M*_neutral_ = 5.44 ± 0.70; *F* (2, 87) = 122.80, *p* < 0.001), but were consistent in arousal (*M*_high_ = 5.96 ± 0.21; *M*_mild_ = 5.88 ± 0.42; *M*_neutral_ = 5.87 ± 0.22; *F* (2, 87) = 1.23, *p* = 0.39). Similarly, deviant images used in the negative block were significantly different in valence (*M*_high_ = 1.81 ± 0.34; *M*_mild_ = 3.62 ± 0.32; *M*_neutral_ = 5.54 ± 0.29; *F* (2, 87) = 1023.20, *p* < 0.001), but consistent in arousal (*M*_high_ = 5.56 ± 0.22; *M*_mild_ = 5.46 ± 0.34; *M*_neutral_ = 5.47 ± 0.40; *F* (2, 87) = 0.88, *p* = 0.42). All images were standardized for size, brightness, and contrast.

### 2.4 Experimental Procedure

**FIGURE 1:**
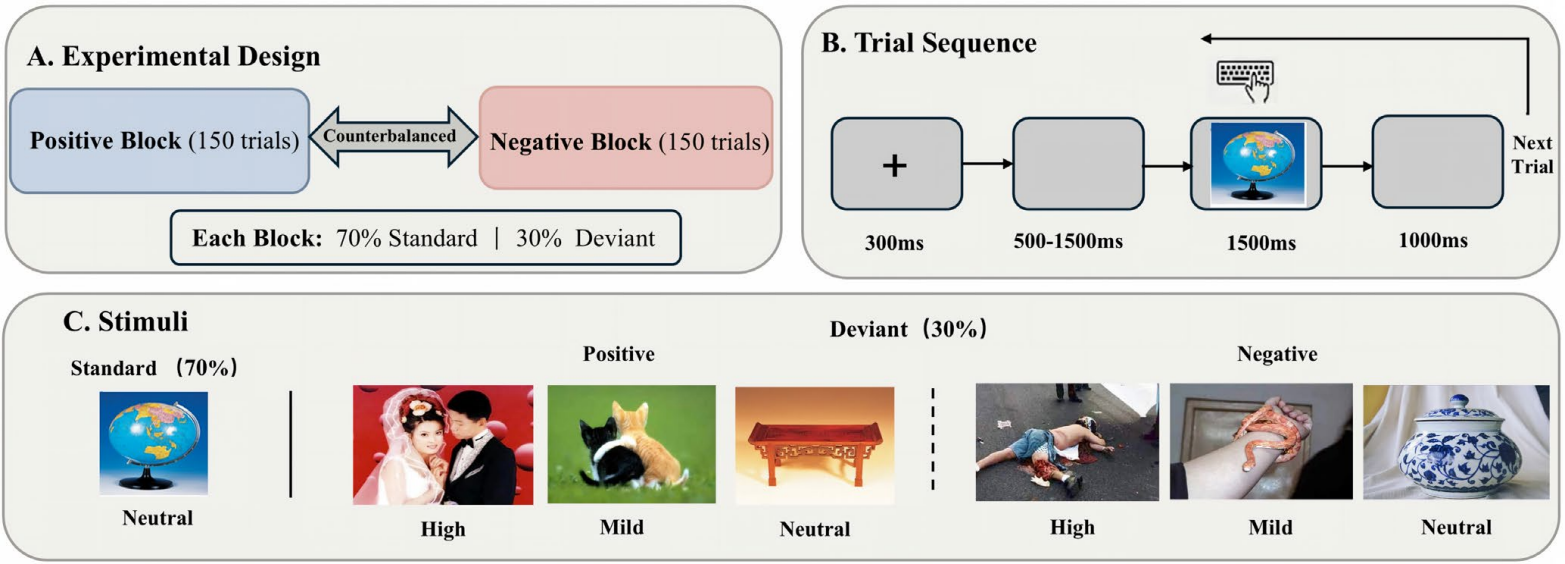
Flow chart of the two‐choice oddball task. (A) Experimental design: A 2 × 2 × 3 mixed factorial design featuring counterbalanced emotional blocks. (B) Trial sequence: Schematic representation of a single trial, including fixation, jittered inter‐stimulus interval (ISI), and target presentation for behavioral identification. (C) Stimuli: Illustration of standard (neutral, 70%) and deviant stimuli (emotional, 30%) stratified by valence and intensity levels.

A two‐choice oddball paradigm was employed [33] (Figure 1). The experiment consisted of two blocks (positive and negative valence), with each containing 150 trials with a 70% standard and 30% deviant probability. The order of the positive and negative blocks was counterbalanced across participants. Each trial started with a 300 ms fixation, followed by a jittered inter‐stimulus interval of 500–1500 ms. The target stimulus was subsequently presented for 1500 ms, during which participants identified the stimulus valence via a keypress. A 1000 ms inter‐trial interval followed. Response hands were also counterbalanced across participants.

### 2.5 EEG Data Analysis

#### 2.5.1 EEG Preprocessing

EEG data were acquired using an 88‐channel ANT system (1000 Hz sampling rate) and analyzed via MATLAB and the EEGLAB toolbox [34]. Preprocessing involved band‐pass filtering (0.5–100 Hz), mastoid re‐referencing, and Independent Component Analysis (ICA) for ocular artifact correction [35]. Data were segmented into epochs from −500 to 1500 ms relative to stimulus onset, with a conservative artifact rejection threshold of ±100 μV applied. Alternative preprocessing parameters employed for supplementary time‐frequency and ERP analyses are detailed in Supporting Informations S1.

#### 2.5.2 PAC Analysis

PAC was quantified using the Kullback–Leibler Modulation Index (KLMI). Phase information was extracted from low‐frequency bands (delta: 1–4 Hz; theta: 4–8 Hz) and amplitude from the beta band (13–30 Hz) via the Hilbert transform, targeting established emotion and vigilance markers while avoiding gamma‐band myogenic artifacts [24]. To mitigate multiple comparison problems [36, 37], analyses were conducted on 20 representative electrodes covering frontal (Fz, F3, F4, F5, F6), central (Cz, C3, C4), parietal (Pz, P3, P4, P5, P6), temporal (T7, T8, TP7, TP8), and occipital regions (Oz, O1, O2). Statistical significance was established using a non‐parametric permutation test with 100 surrogate iterations, and the resulting modulation indices were z‐scored. Only electrode‐frequency pairs exhibiting significant coupling (*p* < 0.05) were retained for subsequent analysis.

#### 2.5.3 Time‐Frequency and ERP Analysis

Time‐frequency representations were computed using complex Morlet wavelets [38, 39], with power normalized to a pre‐stimulus baseline (−300 to 0 ms). The time windows for each frequency band (delta, theta, alpha, and beta) were initially referred to prior literature [40, 41] and subsequently refined by visually inspecting the grand average data collapsed across all conditions to capture peak activity. To maximize the signal‐to‐noise ratio, these oscillatory activities were extracted from specific anterior (F1, Fz, F2) and posterior (P1, Pz, P2) midline clusters. Additionally, standard ERP components were extracted as supplementary measures.

### 2.6 Statistical Analysis

Linear mixed‐effects modeling (LMM) was performed for data analysis using R (version 4.5.0) with the lme4 package. The assumptions of normality and homogeneity of variance for the linear mixed‐effects models were verified by visually inspecting the Q‐Q plots and residual plots, complemented by the Shapiro–Wilk test. The analyses confirmed that the model residuals followed a Gaussian distribution. Two participants from the NCID group and one participant from the NC group were excluded due to excessive EEG artifacts (overall rejection rate > 20%), resulting in a final sample of 44 participants for formal data analysis, with an average trial retention rate of ~85% (minimum 80%). For behavioral indicators, LMM analyses were employed for performance indicators of accuracy and reaction time (RT), with Group, Valence, and Intensity as fixed factors and participants as random intercepts.

For PAC analysis, two complementary LMMs were constructed using normalized KLMI values as the dependent variables. The first model assessed CFC differences between groups across emotional conditions with Group, Valence, and Intensity as fixed factors, Subject and Channel as nested random intercepts to account for individual variability and electrode dependencies. The second model examined differences across five brain regions (frontal, central, parietal, temporal, and occipital) with Group, Brain Region, and Valence as fixed factors. Statistical significance was determined using type III Wald chi‐square tests, with post hoc comparisons using Bonferroni‐adjusted estimated marginal means (EMMs). For clarity, all PAC values were multiplied by 10^4^ in the results section and figures.

For time‐frequency analysis, the LMMs with Group, Valence, Intensity, and Brain region (anterior, posterior regions) as fixed factors and with ERSP values as the dependent variable (analyzed separately for delta, theta, alpha, and beta bands). For supplementary ERP analysis, component amplitudes (N2, P2, LPP, and EPN) were similarly modeled with component‐specific electrode clusters. Three complementary strategies were employed to rigorously control for Type I errors and multiple comparisons: (1) significance for CFC was established using non‐parametric permutation testing with 100 surrogate iterations [37]; (2) LMMs provided natural regularization through partial pooling, significantly reducing false positive rates [42]; and (3) all post hoc pairwise comparisons were strictly adjusted using the Bonferroni corrections. Effect sizes were reported as *Cohen's d* (between‐group comparisons) and partial eta‐squared ($\eta_{p}^{2}$) for interactions, with a significance threshold at *p* < 0.05. Comprehensive statistical details are provided in the Supporting Informations.

## 3 Results

### 3.1 Demographic and Baseline Characteristics

Demographic and baseline psychological characteristics are presented in Table 1. The NC and NCID groups showed no significant differences in age, sex distribution, or education (all *p* > 0.05). However, compared with the NC group, the NCID group exhibited significantly higher scores on sleep disturbance (PSQI, ISI), depressive symptoms (BDI‐II), negative mood, and emotion regulation difficulties (Difficulties in Emotion Regulation Scale, DERS) (all *p* < 0.05).

**TABLE 1:** Baseline comparison of sleep quality and mood between two groups.

	NC (*n* = 22)	NCID (*n* = 25)	Statistics
Age (years)	20.54 ± 2.32	21.76 ± 2.14	*t* = −1.88, *p* = 0.070
Female, *n* (%)	14 (63.6%)	20 (80.0%)	*χ* ^2^ = 1.57, *p* = 0.210
Education (years)	14.80 ± 1.20	15.11 ± 1.40	*t* = −0.78, *p* = 0.440
PSQI	4.85 ± 1.52	10.48 ± 2.18	*t* = −10.13, *p* < 0.001
ISI	3.23 ± 1.84	12.36 ± 3.12	*t* = −12.00, *p* < 0.001
BDI‐II	1.15 ± 2.23	7.00 ± 2.22	*t* = −8.13, *p* < 0.001
Positive mood	35.26 ± 6.31	28.47 ± 6.42	*t* = 0.71, *p* = 0.640
Negative mood	16.31 ± 6.12	22.36 ± 6.98	*t* = −3.42, *p* = 0.030
DERS	44.52 ± 6.83	57.63 ± 7.72	*t* = −3.91, *p* = 0.020

### 3.2 Behavioral Performance

Descriptive statistics for accuracy and reaction time across all conditions are presented in Table 2. Model assumptions of normality and variance homogeneity were verified using Q‐Q and residual plots alongside the Shapiro‐Wilk test (Accuracy: W = 0.94, *p* < 0.001; RT: W = 0.89, *p* < 0.001).

**TABLE 2:** Behavioral performance (Accuracy and RT) across experimental conditions.

Valence	Intensity	NC (*n* = 22)		NCID (*n* = 25)	
		Accuracy (%)	RT (ms)	Accuracy (%)	RT (ms)
Positive	High	96.70 ± 3.85	459.00 ± 64.40	98.70 ± 2.33	510.00 ± 142.00
	Mild	97.30 ± 3.44	460.00 ± 65.40	98.30 ± 2.36	531.00 ± 133.00
	Neutral	99.30 ± 1.41	445.00 ± 63.90	99.70 ± 1.05	522.00 ± 170.00
Negative	High	97.00 ± 6.18	442.00 ± 48.10	98.70 ± 1.72	502.00 ± 81.50
	Mild	94.30 ± 5.68	458.00 ± 49.20	98.00 ± 2.33	515.00 ± 96.50
	Neutral	98.30 ± 4.23	435.00 ± 56.10	98.30 ± 1.76	511.00 ± 103.00

LMM for accuracy revealed a significant main effect of Intensity [*F* (2, 230) = 7.53, *p* = 0.001, $\eta_{p}^{2}=0.057$], with higher accuracy observed for high and mild intensity images compared to neutral images (EMM_high_ = 0.97, SE = 0.005, *p* = 0.007; EMM_mild_ = 0.98, SE = 0.005, *p* = 0.001; EMM_neutral_ = 0.96, SE = 0.005).

LMM for RT revealed significant main effects of Group and Intensity [*F* (1, 46) = 7.13, *p* = 0.010, $\eta_{p}^{2}=0.026$; *F* (2, 230) = 8.34, *p* < 0.001, $\eta_{p}^{2}=0.026$], with longer RTs being elicited in the NCID versus NC group (543 ± 20.0 vs. 467 ± 20.9). The Group × Valence interaction effect was significant [*F* (1, 230) = 8.32, *p* = 0.004, $\eta_{p}^{2}$ = 0.031]. Post hoc analyses showed that the NCID group responded significantly faster to negative images than to positive ones (EMM_positive_ = 555, SE = 20.3; EMM_negative_ = 531, SE = 20.3, *p* < 0.001), while no such valence‐dependent difference was observed in the NC group (*p* = 0.306).

### 3.3 PAC Indicators

The comprehensive statistical outcomes for all main effects and interactions of the PAC analyses are presented in Table 3 and S2. The raw data points for the regional PAC analysis are provided in Table S1. Model assumptions were verified using Q‐Q and residual plots alongside the Shapiro‐Wilk test (Delta‐Beta PAC: W = 0.99, *p* < 0.001; Theta‐Beta PAC: W = 0.98, *p* < 0.001).

**TABLE 3:** Linear mixed effects model results for phase amplitude coupling.

Source of variance	df (Delta‐Beta)	*F*	*p*	$\eta_{p}^{2}$	df (Theta‐Beta)	*F*	*p*	$\eta_{p}^{2}$
Group	1, 9311	23.49	< 0.001	0.005	1, 50,981	68.29	< 0.001	0.015
Valence	1, 4300	613.71	< 0.001	0.124	1, 50,981	134.33	< 0.001	0.029
Intensity	2, 4300	81.08	< 0.001	0.036	2, 50,981	46.59	< 0.001	0.02
Brain Region	4, 1136	286.7	< 0.001	0.206	4, 51	318.13	< 0.001	0.204
Group × Valence	1, 4300	0.12	0.732	0.001	1, 50,981	115.11	< 0.001	0.025
Group × Intensity	2, 4300	17.92	< 0.001	0.008	2, 50,981	108.28	< 0.001	0.046
Valence × Intensity	2, 4300	61.58	< 0.001	0.028	2, 50,981	87.67	< 0.001	0.038
Group × Brain Region	4, 1136	40.63	< 0.001	0.035	4, 51	13.17	< 0.001	0.01
Brain Region × Valence	4, 4300	44.39	< 0.001	0.039	4, 377	4.88	0.001	0.004
Group × Valence × Intensity	2, 4300	68.45	< 0.001	0.031	2, 50,981	72.42	< 0.001	0.031
Group × Brain Region × Valence	4, 4300	96.5	< 0.001	0.08	4, 377	10.24	< 0.001	0.008

**FIGURE 2:**
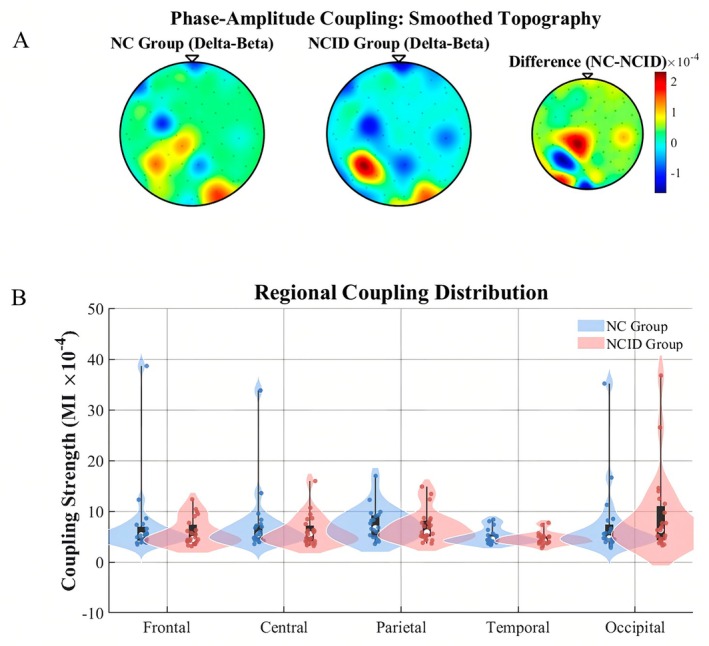
Spatial distribution and regional analysis of delta‐beta phase‐amplitude coupling (PAC). (A) Topographic maps of mean delta‐beta coupling strength (Modulation Index, MI) for the NC and NCID groups, alongside the spatial difference (NC minus NCID). Warmer colors indicate stronger coupling. (B) Regional coupling strength distribution. Violin plots illustrate the delta‐beta coupling across five regions of interest. Blue (NC) and red (NCID) violins display the kernel density estimation of the data. The blank dots represent exact data points for each subject, and internal box plots indicate the median and interquartile range. All MI values are ×10^4^.

LMM analysis showed distinct group differences for the two coupling types (Figure 2). For delta‐beta coupling (values ×10^4^), the NCID group exhibited significantly lower global coupling values compared to the NC group (EMM_NC_ = 7.5, SE = 0.1; EMM_NCID_ = 7.0, SE = 0.1, *p* < 0.001). Conversely, for theta‐beta coupling, the NCID group showed significantly higher coupling values than the NC group (EMM_NCID_ = 1.4, SE < 0.001; EMM_NC_ = 1.3, SE < 0.001, *p* < 0.001).

A significant Group × Brain Region interaction was revealed for both coupling types. For delta‐beta coupling, the NCID group showed significantly lower coupling values in frontal (EMM_NCID_ = 6.3 vs. EMM_NC_ = 7.9, *p* < 0.001) and central regions (EMM_NCID_ = 6.6 vs. EMM_NC_ = 8.2, *p* < 0.001) compared to the NC group. For theta‐beta coupling, the NCID group exhibited higher coupling in occipital (EMM_NCID_ = 1.8 vs. EMM_NC_ = 1.6, *p* < 0.001) and central regions (EMM_NCID_ = 1.4 vs. EMM_NC_ = 1.2, *p* < 0.001) relative to the NC group.

For delta‐beta coupling, Intensity and Group × Intensity induced significant effects; the post hoc analysis revealed that the high intensity images induced higher coupling than neutral images in both the NC group (EMM_high_ = 7.6, SE = 0.1; EMM_mild_ = 7.2, SE = 0.1; EMM_neutral_ = 6.9, SE = 0.1, *p* < 0.001) and NCID group (EMM_high_ = 7.4, SE = 0.1; EMM_mild_ = 6.9, SE = 0.1; EMM_neutral_ = 6.7, SE = 0.1, *p* < 0.001), yet such difference was more pronounced in NC group.

For theta‐beta coupling, the Group × Intensity interaction reached significance. The NC group exhibited lower coupling for high‐intensity versus neutral images (*p* < 0.001), while the NCID group showed higher coupling for neutral images compared to high (*p* < 0.001) and mild intensity images (*p* < 0.001).

Valence induced a significant main effect on both the delta‐beta and theta‐beta coupling, with negative images eliciting higher coupling than positive images. Post hoc comparisons for the three‐way Group × Valence × Intensity interaction revealed that under the high‐intensity negative condition, the NCID group exhibited significantly lower delta‐beta coupling than the NC group (7.6 ± 0.1 vs. 8.4 ± 0.1, *p* < 0.001) and neutral‐intensity positive images (6.4 ± 0.1 vs. 7.1 ± 0.1, *p* < 0.001), whereas no significant group difference was observed for neutral images in negative block (*p* = 0.572). For theta‐beta coupling, the NCID group showed significantly higher coupling for mild‐intensity negative images compared to the NC group (1.5 ± 0.001 vs. 1.3 ± 0.001, *p* < 0.001) and high‐intensity images regardless of valence (all *p* < 0.001).

### 3.4 Time‐Frequency Domain Indicators

**FIGURE 3:**
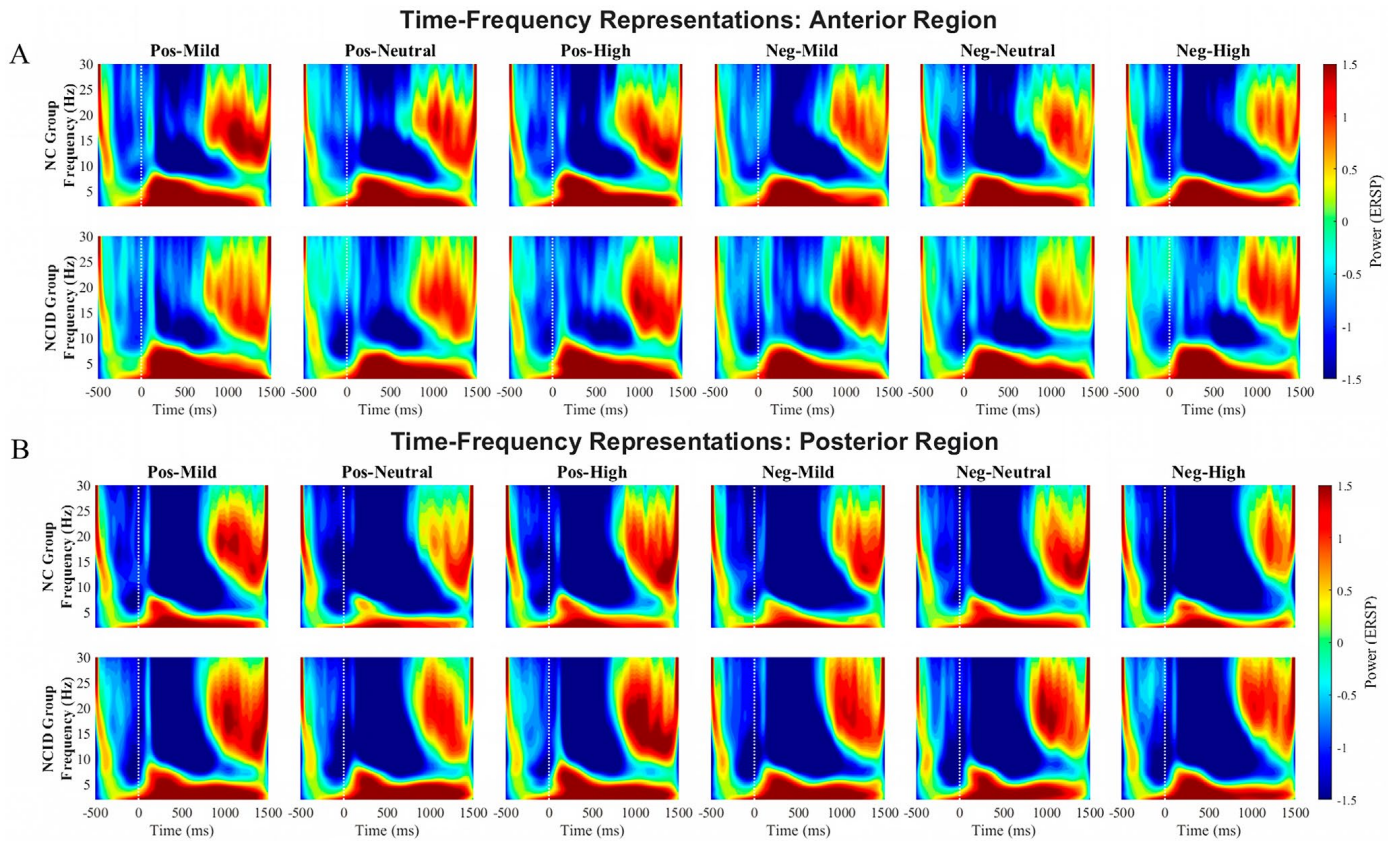
Group‐averaged results of evoked EEG energy in anterior and posterior regions across conditions. (A) Event‐related spectral perturbation (ERSP) maps in the anterior region (averaged over F1, Fz, and F2). (B) ERSP maps in the posterior region (averaged over P1, Pz, and P2). The white dotted line at 0 ms indicates the onset of the visual stimulus.

Model assumptions for time frequency analyses were verified using Q‐Q and residual plots alongside the Shapiro Wilk test with W values ranging from 0.993 to 0.998 (*p*s < 0.05). Time‐frequency analysis corroborated the PAC findings with robust oscillatory differences. Generally, lower‐frequency oscillations (< 8 Hz) exhibited event‐related synchronization (ERS) during the 0–500 ms window, while higher‐frequency bands showed event‐related desynchronization (ERD) in the 500–1000 ms period (see Figure 3).

Group induced a significant main effect on the theta band [*F* (1, 41) = 5.73, *p* = 0.021, $\eta_{p}^{2}=0.123$], with the NCID group exhibiting greater global theta power. The Group × Brain Region interaction localized this effect to posterior regions [*F* (1, 451) = 23.93, *p* < 0.001, $\eta_{p}^{2}=0.050$], where the NCID group showed significantly higher theta power compared to NC group (0.83 ± 0.20 vs. −0.24 ± 0.22, *p* < 0.001). Similarly, the delta band showed a significant interaction [*F* (1, 451) = 11.19, *p* < 0.001, $\eta_{p}^{2}=0.024$], with the NCID group showed marginally greater posterior delta power (1.49 ± 0.13 vs. 1.13 ± 0.13, *p* = 0.054). Regarding beta power, a significant Valence × Intensity interaction revealed that the valence effect varied across emotional intensities [*F* (2, 451) = 4.96, *p* = 0.007, $\eta_{p}^{2}=0.022$]. Specifically, positive images elicited significantly greater beta power than negative images under both mild (−0.67 ± 0.14 vs. −1.03 ± 0.14, *p* < 0.001) and high intensity conditions (−0.80 ± 0.14 vs. −1.19 ± 0.14, *p* < 0.001). However, no significant difference was observed for neutral images.

Complementary time‐domain analyses statistical outcomes are provided in Data S3. Complementary ERP components (N2, P2, LPP, EPN) revealed statistically significant alterations in partial components and detailed results were presented in Data S4 and Figure S1, respectively. In addition, to synthesize the multitude of findings across different domains, a comprehensive overview of the key group differences and condition modulations for both behavioral and neurophysiological metrics is provided in Table 4.

**TABLE 4:** Summary of behavioral and neurophysiological findings.

Domain	Metric	Region of interest	Main group effect (NCID vs. NC)	Key interactions & modulations
Behavior	RT	—	NCID > NC (Slower)	NCID: Negative RT < Positive RT
	ACC	—	None	High & Mild > Neutral
PAC	Delta‐Beta	Frontal, Central	NCID < NC (↓)	NCID: Blunted intensity sensitivity
				NCID < NC (High‐negative)
	Theta‐Beta	Occipital, Central	NCID > NC (↑)	NCID > NC (Mild‐negative)
Time‐Frequency	Theta power	Posterior	NCID > NC (↑)	Positive> Negative overall
	Delta power	Posterior	NCID > NC (Marginal ↑)	—
	Beta power	Anterior > Posterior	None	Positive > Negative

## 4 Discussion

This study examined the neurophysiological basis of emotional susceptibility in insomnia using multimodal EEG measurements. Results indicate that individuals with insomnia exhibit a dual dysregulation pattern characterized by attenuated frontal delta‐beta coupling and potentiated posterior theta‐beta coupling. These neural alterations were not uniform but were modulated by specific combinations of emotional intensity and valence, showing a pronounced vulnerability to negative stimuli. These findings demonstrate that emotional susceptibility in insomnia stems from a fundamental imbalance involving a compromised capacity for top‐down cognitive control and heightened bottom‐up sensory reactivity.

Behaviorally, distinct response profiles emerged between groups. Both groups demonstrated higher accuracy for emotional stimuli, which may be attributed to the automatic attentional prioritization of salient information [43, 44]. However, the NCID group exhibited generally slower RTs, consistent with decreased efficiency in attentional resource allocation among individuals with sleep disturbances [45]. Notably, analysis revealed a valence‐dependent bias in the NCID group, whereby participants responded significantly faster to negative images than to positive ones. This pattern was absent in controls. Unlike previous reports of reduced sensitivity to negative expressions in explicit tasks [14], current findings suggest that sleep disturbance may differentially affect automatic and explicit processing layers. This leads to enhanced unconscious reactivity to negative cues even when conscious appraisal remains blunted.

### 4.1 Dual Dysregulation Pattern of CFC in Insomnia

The observed double dissociation in PAC patterns highlights distinct mechanistic failures in the neural circuitry of insomnia. The significant reduction in delta‐beta coupling within frontal and central regions in the NCID group indicates a deficit in top‐down regulation. Delta‐beta coupling at frontal sites has been linked to regulatory mechanisms [24], its attenuation implies a weakened ability to downregulate emotional responses. This interpretation is consistent with neuroimaging studies reporting hypoactivation of the prefrontal cortex during executive tasks in insomnia [46] and suggests a chronic impairment in the neural substrates required for effortful emotional control.

Conversely, the NCID group exhibited enhanced theta‐beta coupling, particularly in occipital regions. Theta‐beta coupling is intrinsically linked to vigilance and emotional arousal [28, 47], suggesting this enhancement reflects a state of sensory hypervigilance. The regional specificity of these findings, characterized by compromised frontal control versus exaggerated posterior processing, aligns at the sensor level with the theoretical framework that emotional regulation relies on dynamic prefrontal‐limbic interactions [48]. In insomnia, this coordination appears to be disrupted, resulting in a system that is overly sensitive to sensory inputs yet ill‐equipped to regulate the resulting downstream activation.

### 4.2 Intensity‐Dependent and Valence‐Specific Alterations

Neural anomalies in insomnia were found to be intensity‐dependent. NC group exhibited a graded delta‐beta response sensitive to intensity variations [13]. In contrast, while the NCID group maintained some intensity‐dependent differentiation, the magnitude of this dynamic range was significantly blunted compared to controls. This attenuated neural flexibility suggests a rigid emotional response system that struggles to optimally scale activation according to varying situational demands. Furthermore, the elevated theta‐beta coupling for neutral stimuli in the NCID group supports the hyperarousal hypothesis [15], indicating that the insomnia brain maintains a high baseline level of vigilance regardless of stimulus salience.

These intensity‐dependent deficits were compounded by valence‐specific biases. The NCID group exhibited significantly lower delta‐beta coupling specifically for high‐intensity negative stimuli. This suggests regulatory mechanisms collapse precisely when processing highly salient negative information [49]. Simultaneously, the enhanced theta‐beta coupling for negative stimuli in the NCID group mirrors cognitive models describing selective attention toward threat‐related cues [50]. This specific combination of impaired regulation for high‐intensity negative emotion and heightened detection of negative valence provides a robust neurophysiological explanation for the increased risk of depression and anxiety observed in insomnia populations [8].

### 4.3 Time‐Frequency Indicators

Time‐frequency analysis supported the CFC findings by revealing distinct oscillatory signatures. The NCID group exhibited greater delta and theta power in posterior regions across emotional conditions. Since posterior delta and theta oscillations are involved in motivational processing and salience detection [21, 51], this suggests that additional neural resources are recruited to process salient emotional information in insomnia, regardless of valence. Furthermore, the elevated posterior alpha power observed in the NCID group aligns with the vigilance hypothesis, likely reflecting heightened system activation and the active inhibition of irrelevant inputs to maintain a state of generalized hyperarousal [41, 52]. This convergent evidence from PAC, time‐frequency, and ERP analyses identifies compromised emotional susceptibility as a core neurophysiological vulnerability. This mechanism likely underlies the bidirectional relationship between sleep disturbances and affective disorders, contributing to the increased risk of psychopathology in insomnia.

The observed dual dysregulation provides a translational framework for precision medicine in sleep disorders. Specifically, the attenuation of frontal delta‐beta coupling highlights a viable target for non‐invasive neuromodulation. Transcranial alternating current stimulation (tACS) strategies could be optimized to entrain frontal phase synchronization and restore top‐down cognitive control. In parallel, neurofeedback interventions targeting the downregulation of occipital theta‐beta hyperconnectivity may prove effective in alleviating sensory hyperarousal. Consequently, these frequency‐specific signatures offer a neurophysiological basis for developing phenotype‐based neuromodulatory protocols to address emotional dysfunction in insomnia.

Several limitations should be acknowledged. First, the oddball paradigm primarily assessed implicit emotional processing. Future studies should incorporate explicit tasks to fully characterize the dissociation between conscious and unconscious processing. Second, the use of a subclinical sample warrants caution when generalizing results to clinical insomnia patients. Third, sensor‐level analysis assessed coupling strength but could not resolve precise anatomical generators or the direction of information flow. Given that directional connectivity in scalp EEG is fundamentally limited by volume conduction, future studies utilizing intracranial recordings or EEG‐fMRI are required to map these underlying causal dynamics.

## 5 Conclusion

This study demonstrates that emotional susceptibility in insomnia is characterized by systemic neural dysregulation. Specifically, attenuated frontal delta‐beta coupling reflects impaired cognitive control, whereas potentiated occipital theta‐beta coupling indicates heightened sensory reactivity. These aberrations are not static but are intricately modulated by emotional intensity and valence. These findings clarify the neurophysiological mechanisms linking sleep disturbances to emotional dysfunction and highlight CFC as a potential biomarker for identifying individuals at risk for comorbid affective disorders.

## Funding

This work was supported by the Ministry of Education Research Project of Humanities and Social Sciences (24YJCZH239) Guangdong Provincial Key Laboratory of Optical Information Materials and Technology (2023B1212060065). China Postdoctoral Science Foundation (2025M773445). The Open Project of the Provincial Key Laboratory of Multi‐modal Perception and Intelligent Systems (MPIS202410). Guangdong Provincial Philosophy and Social Sciences Planning (Youth Project, GD26YXL06). Young Teacher Research Cultivation Fund of South China Normal University (24KJ27).

## Ethics Statement

The experimental protocol was established in accordance with the ethical guidelines of the Helsinki Declaration and approved by the Ethics Committee of South China Normal University (SCNU‐PSY‐2022‐241).

## Consent

All participants provided written informed consent before participating in the study.

## Conflicts of Interest

The authors declare no conflicts of interest.

## Supporting information


**Figure S1:** ERP waveforms during emotional processing stages. A, N2 component, waveforms represent the averaged data from F3, F4, Fz, FC1, and FC2 electrode sites. B, P2 component, waveforms represent the averaged data from PO7, PO8, POz, PO5, and PO6 electrode sites. C, LPP component, waveforms represent the averaged data from FC5, FC6, FCz, F7, and F8 electrode sites. D, EPN component, waveforms represent the averaged data from PO7, PO8, P7, P8, O1, and O2 electrode sites.
**Table S1:** Raw data points of phase‐amplitude coupling for regional analysis.

## Data Availability

The data that support the findings of this study are available from the corresponding authors upon reasonable request.
