# Supplementary material for: Decreased Control and Enhanced Reactivity: Dual Dysregulation Pattern of Cross‐Frequency Coupling in Emotional Susceptibility of Non‐Clinical Insomnia
Source: CNS Neurosci Ther. 2026 Jul 29;32(8):e71031. doi: 10.1002/cns.71031 (PMC13418374; doi:10.1002/cns.71031)
Supplement: Supplementary file 1 — Figure S1: ERP waveforms during emotional processing stages. A, N2 component, waveforms represent the averaged data from F3, F4, Fz, FC1, and FC2 electrode sites. B, P2 component, waveforms represent the averaged data from PO7, PO8, POz, PO5, and PO6 electrode sites. C, LPP component, waveforms represent the averaged data from FC5, FC6, FCz, F7, and F8 electrode sites. D, EPN component, waveforms represent the averaged data from PO7, PO8, P7, P8, O1, and O2 electrode sites. Table S1: Raw data points of phase‐amplitude coupling for regional analysis. [file CNS-32-e71031-s001.docx]

**S1. Methodological Details**

EEG data were continuously recorded using an 88-channel ANT system (ANT EEGO; Eemagine Medical Imaging Solutions GmbH, Berlin, Germany) with a sampling rate of 1000 Hz. The electrode montage followed the extended international 10-20 system, referenced online to CPz with the ground at Fpz-Fz. Impedances were maintained below 5 kΩ throughout the recording. Preprocessing pipelines were tailored to specific analytic domains to optimize signal-to-noise ratios. For the primary Phase-Amplitude Coupling (PAC) analysis, continuous data underwent zero-phase band-pass filtering (0.5–100 Hz, 2nd order Butterworth) to preserve high-frequency amplitude modulations, and data were segmented into epochs from -500 to 1500 ms relative to stimulus onset. In contrast, supplementary ERP analysis employed a narrower band-pass filter (0.01–30 Hz) with shorter epochs (-200 to 800 ms) and a stricter artifact rejection threshold (±75 μV) compared to the ±100 μV used for PAC. For time-frequency analysis, epochs were extended (-1000 to 2000 ms) to mitigate edge effects during wavelet convolution, with baseline correction applied from -500 to -200 ms. Ocular artifacts were corrected across all pipelines using Independent Component Analysis (ICA).

Quantification PAC was calculated using the Kullback-Leibler Modulation Index (KLMI). The instantaneous phase of low-frequency oscillations (*f*_P_: delta 1–4 Hz, theta 4–8 Hz) and the amplitude envelope of high-frequency oscillations (*f*_A_: beta 13–30 Hz) were extracted via the Hilbert transform. The composite signal was analyzed across 18 equidistant phase bins (*N*=18). The Modulation Index (MI) was derived using the formula MI = (H _max_ - H) / H _max_ represents the Shannon entropy of the amplitude distribution, and $H= -\sum_{j=1}^{N} p_{j}logp_{j}$ represents the entropy of a uniform distribution. Statistical significance was determined via permutation testing with 100 iterations of time-spliced surrogate data. A normalized z-score was computed for each electrode-frequency pair as ${MI}_{norm}=\frac{{MI}_{obs}-\mu_{surr}}{\sigma_{surr}}$. Analysis was restricted to twenty representative electrodes grouped into five regions: Frontal (Fz, F3, F4, F5, F6), Central (Cz, C3, C4), Parietal (Pz, P3, P4, P5, P6), Temporal (T7, TP7, TP8), and Occipital (Oz, O1, O2).

Time-frequency power was computed using a Morlet continuous wavelet transform with a center frequency (ω) of 5 and constraint (σ) of 0.15, covering 1–30 Hz in 0.5 Hz steps. Power values were decibel-normalized relative to the pre-stimulus baseline. Regions of interest were defined for delta (2–4 Hz, 100–300 ms), theta (4–7 Hz, 300–1000 ms), alpha (8–12 Hz, 100–650 ms), and beta (13–30 Hz, 250–850 ms) bands. For ERP analysis, component amplitudes were extracted from specific time windows and electrode clusters: N2 (160–260 ms at F3, F4, Fz, FC1, FC2), P2 (120–250 ms at PO7, PO8, POz, PO5, PO6), LPP (300–600 ms at FC5, FC6, FCz, F7, F8), and EPN (200–300 ms at PO7, PO8, P7, P8, O1, O2). The rationale for employing different spatial regions depends on the distinct neural dynamics measured. PAC indexes large-scale network communication (Canolty & Knight, 2010); thus, grouping electrodes into five broad macroscopic regions (frontal, central, parietal, temporal, and occipital) is a standard approach to capture global coordination while managing multiple comparisons (Cohen, 2014). Conversely, task-evoked time-frequency power (ERSP) during emotion processing elicits highly localized focal responses (Güntekin & Basar, 2014). Averaging across broad scalp divisions would dilute this signal. Therefore, time-frequency analyses were restricted to specific anterior and posterior midline clusters to maximize the signal-to-noise ratio of these localized dynamics.

**S2. PAC Results**

**S2.1 Complete Regional Analysis of PAC**

A robust main effect of Brain Region was observed [*F* (4, 1136) = 286.70, *η2 p* = 0.206, *p* < 0.001]. Post-hoc comparisons revealed the highest PAC values in the occipital region (EMM = 8.8, *SE* = 0.1), followed by parietal (EMM = 7.9, *SE* = 0.1), central (EMM = 7.4, *SE* = 0.1), frontal (EMM = 7.1, *SE* = 0.1), and temporal regions (EMM = 5.4, *SE* = 0.1). All pairwise comparisons between regions were significant (*p*s < 0.05) except between central and frontal regions in the NC group (*p* = 0.552).

A significant Brain Region × Valence interaction was found [*F* (4, 4300) = 44.39, *η2 p* = 0.039, *p* < 0.001]. The valence effect (negative > positive) was most pronounced in the occipital region (difference = 1.1, *p* < 0.001), followed by central (difference = 0.7, *p* < 0.001), frontal (difference = 0.5, *p* < 0.001), parietal (difference = 0.3, *p* < 0.001), and temporal regions (difference = 0.2, *p* < 0.001).

A robust main effect of Brain Region was observed [*F* (4, 51) = 318.13, *η2 p* = 0.204, *p* < 0.001]. Post-hoc comparisons indicated significantly higher PAC values in the occipital region (EMM = 1.7, *SE* < 0.001) compared to all other regions. The temporal region showed the second highest PAC values (EMM = 1.4, *SE* < 0.001), followed by parietal (EMM = 1.3, *SE* < 0.001), while frontal and central regions exhibited similar and the lowest PAC values (EMM _frontal_ = 1.3, *SE* < 0.001; EMM _central_ = 1.3, *SE* < 0.001, *p* = 1.000).

A significant Brain Region × Valence interaction was also found [*F* (4, 377) = 4.88, *η2 p* = 0.004, *p* = 0.001]. The valence effect (negative > positive) was most pronounced in the temporal region (difference = 0.1, *p* < 0.001), followed by the central region (difference = 0.1, *p* < 0.001), and was least evident in the frontal region (difference < 0.001, *p* = 0.027).

**S2.2 Complete Intensity Analysis of PAC**

A significant main effect of Intensity was observed [*F* (2, 4300) = 81.08, *η2 p* = 0.036, *p* < 0.001]. Post-hoc comparisons revealed that high intensity images elicited the highest PAC, followed by low intensity images, with neutral intensity images showing the lowest PAC values (EMM _high_ = 7.6, *SE* = 0.1; EMM _low_ = 7.2, *SE* = 0.1; EMM _neutral_ = 6.9, *SE* = 0.1). All pairwise comparisons were significant (*p*s < 0.001).

A significant Group × Intensity interaction was observed [*F* (2, 4300) = 17.92, *η2 p* = 0.008, *p* < 0.001]. In the NC group, the difference between high and neutral intensity images (difference = -0.8, *p* < 0.001) was larger than the same comparison in the NCID group (difference = -0.7, *p* < 0.001).

A significant Valence × Intensity interaction was also found [*F* (2, 4300) = 61.58, *η2 p* = 0.028, *p* < 0.001]. For negative images, the difference between high and neutral intensity stimuli (difference = -1.0, *p* < 0.001) was more pronounced than for positive images (difference = -0.5, *p* < 0.001).

A significant three-way interaction among Group, Valence, and Intensity was detected [*F* (2, 4300) = 68.45, *η2 p* = 0.031, *p* < 0.001]. Post-hoc analyses revealed that for high intensity negative images, the NC group showed significantly higher PAC values (EMM = 8.4, *SE* = 0.1) compared to the NCID group (EMM = 7.6, *SE* = 0.1, *p* < 0.001). For neutral intensity positive images, the NC group also exhibited significantly higher PAC values (EMM = 7.1, *SE* = 0.1) than the NCID group (EMM = 6.4, *SE* = 0.1, *p* < 0.001). Interestingly, for neutral intensity negative images, there was no significant difference between groups (*p* = 0.572).

A significant main effect of Intensity was observed [*F* (2, 50981) = 46.59, *η2 p* = 0.020, *p* < 0.001]. Post-hoc comparisons indicated that neutral intensity images elicited the highest PAC values (EMM = 1.4, *SE* < 0.001), which were significantly higher than both low intensity (EMM = 1.4, *SE* < 0.001, *p* < 0.001) and high intensity images (EMM = 1.4, *SE* < 0.001, *p* < 0.001). No significant difference was found between low and high intensity conditions (*p* = 0.801).

A significant Group × Intensity interaction emerged [*F* (2, 50981) = 108.28, *η2 p* = 0.046, *p* < 0.001]. In the NC group, high intensity images elicited the lowest PAC values (EMM = 1.3, *SE* < 0.001), which differed significantly from both low intensity (EMM = 1.3, *SE* < 0.001, *p* < 0.001) and neutral intensity images (EMM = 1.3, *SE* < 0.001, *p* < 0.001). In contrast, the NCID group showed the highest PAC for neutral intensity images (EMM = 1.5, *SE* < 0.001), with significant differences observed across all intensity levels (*p*s < 0.001).

A significant Valence × Intensity interaction was also found [*F* (2, 50981) = 87.67, *η2 p* = 0.038, *p* < 0.001]. For positive images, PAC values were similar between neutral and high intensity conditions (EMM _neutral_ = 1.4, *SE* < 0.001; EMM _high_ = 1.4, *SE* < 0.001, *p* = 1.000), with both being significantly higher than low intensity (EMM = 1.3, *SE* = 0.0, *p*s < 0.001). For negative images, a different pattern emerged, with neutral intensity images showing the highest PAC (EMM = 1.4, *SE* < 0.001), followed by low intensity (EMM = 1.4, *SE* < 0.001), and high intensity showing the lowest values (EMM = 1.3, *SE* < 0.001). All pairwise comparisons for negative images were significant (*p*s < 0.001).

A highly significant three-way interaction among Group, Valence, and Intensity was observed [*F* (2, 50981) = 72.42, *η2 p* = 0.031, *p* < 0.001]. Post-hoc analyses showed that group differences were most pronounced for negative low intensity images, with the NCID group exhibiting significantly higher PAC values (EMM = 1.5, *SE* < 0.001) compared to the NC group (EMM = 1.3, *SE* < 0.001, *p* < 0.001). Interestingly, for positive low intensity images, this pattern was reversed, with the NC group showing slightly higher PAC values (EMM = 1.3, *SE* < 0.001) than the NCID group (EMM = 1.3, *SE* < 0.001), though this difference only approached significance (*p* = 0.058). For high intensity conditions with both positive and negative valence, the NCID group consistently showed higher PAC values than the NC group (*p*s < 0.001).

**S2.3 Additional Three-Way Interactions**

The three-way interaction among Group, Brain Region, and Valence [*F* (4, 4300) = 96.50, *η2 p* = 0.080, *p* < 0.001] revealed detailed regional patterns. Post-hoc analyses showed that the most pronounced group differences were in the frontal region for negative stimuli (EMM _NC_ = 8.3, *SE* = 0.1; EMM _NCID_ = 6.4, *SE* = 0.1, *p* < 0.001) and in the central region for negative stimuli (EMM _NC_ = 8.7, *SE* = 0.1; EMM _NCID_ = 6.8, *SE* = 0.1, *p* < 0.001). In contrast, for the occipital region, the NCID group showed significantly higher PAC values for negative stimuli (EMM = 10.8, *SE* = 0.1) compared to the NC group (EMM = 7.9, *SE* = 0.1, *p* < 0.001). Notably, for temporal regions with negative valence stimuli, no significant group difference was observed (*p* = 0.096).

The Group × Valence interaction approached significance [*F* (1, 4300) = 3.53, *η2 p* = 0.001, *p* = 0.060]. Both groups showed significantly higher PAC for negative compared to positive images, with the difference being slightly larger in the NCID group (difference = 0.6, *p* < 0.001) compared to the NC group (difference = 0.5, *p* < 0.001).

The three-way interaction among Group, Brain Region, and Valence [*F* (4, 377) = 10.24, *η2 p* = 0.008, *p* < 0.001] showed varied patterns across brain regions. Post-hoc analyses demonstrated that group differences varied markedly across regions and valence conditions. The most striking finding was observed in the occipital region with negative valence stimuli, where the NCID group showed dramatically higher PAC values (EMM = 1.9, *SE* < 0.001) compared to the NC group (EMM = 1.5, *SE* < 0.001, *p* < 0.001). Notably, in the parietal region with positive valence stimuli, no significant group difference was observed (EMM _NCID_ = 1.3, *SE* < 0.001; EMM _NC_ = 1.3, *SE* < 0.001, *p* = 0.165), while with negative valence, the NCID group exhibited significantly higher PAC values (*p* < 0.001).

**S3. Time-Frequency Domain Results**

All twelve conditions (NCID-high positive/negative, NCID-neutral, NCID-low positive/negative, NC-high positive/negative, NC-neutral, NC-low positive/negative) exhibited similar patterns. Lower frequency bands (< 8 Hz) displayed maximum event-related synchronization (ERS) at 0-500 ms, while higher frequency bands showed maximum event-related desynchronization (ERD) at 500-1000 ms (see Figure 3).

For delta band power, a significant main effect of Scalp Distribution was observed [*F* (1, 451) = 31.21, *η2 p* = 0.065, *p* < 0.001], with anterior regions exhibiting greater delta power than posterior regions (EMM _anterior_ = 1.67 ± 0.09, EMM _posterior_ = 1.31 ± 0.09). A significant Group × Scalp Distribution interaction was found [*F* (1, 451) = 11.19, *η2 p* = 0.024, *p* < 0.001]. Post-hoc analyses revealed that in posterior regions, the NCID group showed marginally greater delta power than the NC group (EMM _NCID_ = 1.49 ± 0.13, EMM _NC_ = 1.13 ± 0.13, *p* = 0.054), No significant main effects of Group, Valence, or Intensity were found, nor were there any other significant interactions (*p*s > 0.05).

For theta band power, a significant main effect of Group was observed [*F* (1, 41) = 5.73, *η2 p* = 0.123, *p* = 0.021], with the NCID group exhibiting greater theta power than the NC group (EMM _NCID_ = 1.08 ± 0.20, EMM _NC_ = 0.39 ± 0.21). A significant main effect of Valence was found [*F* (1, 451) = 9.38, *η2 p* = 0.020, *p* = 0.002], with positive images eliciting significantly greater theta power than negative images (EMM _negative_ = 0.62 ± 0.15; EMM _positive_ = 0.86 ± 0.15). A significant main effect of Scalp Distribution was observed [*F* (1, 451) = 132.95, *η2 p* = 0.228, *p* < 0.001], with anterior regions exhibiting greater theta power than posterior regions (EMM _anterior_ = 1.18 ± 0.15, EMM _posterior_ = 0.30 ± 0.15). A significant Group × Scalp Distribution interaction was found [*F* (1, 451) = 23.93, *η2 p* = 0.050, *p* < 0.001]. Post-hoc analyses revealed that in posterior regions, the NCID group showed greater theta power than the NC group (EMM _NCID_ = 0.83 ± 0.20, EMM _NC_ = -0.24 ± 0.22, *p* < 0.001), while no significant group difference was observed in anterior regions (*p* = 0.301). No significant main effect of Intensity or other interactions were observed (*p*s > 0.05).

For alpha band power, a significant main effect of Valence was observed [*F* (1, 451) = 5.22, *η2 p* = 0.011, *p* = 0.023], with positive images eliciting significantly greater alpha power than negative images (EMM _negative_ = -1.21 ± 0.31; EMM _positive_ = -1.00 ± 0.31). A significant main effect of Scalp Distribution was found [*F* (1, 451) = 193.72, *η2 p* = 0.300, *p* < 0.001], with anterior regions exhibiting greater alpha power than posterior regions (EMM _anterior_ =-0.45 ± 0.31, EMM _posterior_ = -1.76 ± 0.31). A significant main effect of Intensity was also observed [F (2, 451) = 3.16, *p* = 0.043, *η2 p* = 0.014]. A significant Group × Scalp Distribution interaction was observed [*F* (1, 451) = 18.83, *η2 p* = 0.040, *p* < 0.001]. Post-hoc analyses revealed that in posterior regions, the NCID group showed greater alpha power than the NC group (EMM _NCID_ = -0.95 ± 0.42, EMM _NC_ =-2.57 ± 0.45, *p* = 0.012), while no significant difference was observed in anterior regions (*p* = 0.199). The main effect of Group was marginally significant [*F* (1, 41) = 3.96, *η2 p* = 0.088, *p* = 0.053]. No other interactions were found (*p*s > 0.05).

For beta band power, a significant main effect of Valence was observed [*F* (1, 451) = 17.53, *η2 p* = 0.037, *p* < 0.001], with positive images eliciting significantly greater beta power than negative images (EMM _negative_ = -1.06 ± 0.13; EMM _positive_ = -0.81 ± 0.13). A significant main effect of Scalp Distribution was found [*F* (1, 451) = 329.53, *η2 p* = 0.422, *p* < 0.001], with anterior regions exhibiting greater beta power than posterior regions (EMM _anterior_ = -0.39 ± 0.13, EMM _posterior_ = -1.47 ± 0.13). A significant Valence × Intensity interaction was observed [*F* (2, 451) = 4.96, *η2 p* = 0.022, *p* = 0.007]. Under low and high intensity conditions, positive images elicited greater beta power than negative images (low intensity: EMM _negative_ = -1.03 ± 0.14, vs. EMM _positive_ = -0.67 ± 0.14, *p* < 0.001; high intensity: EMM _negative_ = -1.19 ± 0.14 vs. EMM _positive_ = -0.80 ± 0.14, *p* < 0.001), while no significant difference was found under neutral intensity conditions (*p* = 0.883). No significant main effects of Group or Intensity, nor any other significant interactions were observed (*p*s > 0.05).

**S4. ERP Results**

For time-domain analysis, ERP components were extracted from the oddball task data as a supplementary measure. Trials were averaged by valence intensity levels to obtain subject-level ERPs, which were then averaged to produce group-level waveforms. Component identification was based on established time windows and electrode clusters: N2 (160-260 ms; F3, F4, Fz, FC1, FC2), P2 (120-250 ms; PO7, PO8, POz, PO5, PO6), LPP (300-600 ms; FC5, FC6, FCz, F7, F8), and EPN (200-300 ms; PO7, PO8, P7, P8, O1, O2). Peak amplitudes were extracted for N2 and P2, while mean amplitudes were calculated for LPP and EPN.

For the N2 component peak, a significant main effect of Group was observed [*F* (1, 43) = 10.02, *η2 p*= 0.038, *p* = 0.003]. The NCID group exhibited significantly smaller N2 amplitude than the NC group (EMM _NCID_ = -0.12, *SE* = 0.91, EMM _NC_ = -4.25, *SE* = 0.97, *p* = 0.003). A significant Group × Valence interaction was found [*F* (1, 215) = 12.13, *η2 p*= 0.047, *p* = 0.001]. In the NCID group, negative images elicited greater N2 amplitude than positive images (EMM _positive_ = 0.79, *SE* = 0.94; EMM _negative_ = -1.03, *SE* = 0.94, *p* < 0.001), while no significant difference was observed in the NC group (*p* = 0.28). A significant Valence × Intensity interaction was observed [*F* (2, 215) = 3.58, *η2 p*= 0.028, *p* = 0.030]. For negative images, high intensity images elicited greater N2 amplitude than low intensity images (EMM _low_ = -1.68, *SE* = 0.99; EMM _high_ = -3.87, *SE* = 0.99, *p* = 0.005), while no significant difference was found for neutral intensity images (*p* > 0.05). For positive images, low intensity images elicited greater N2 amplitude than neutral intensity images (EMM _neutral_ = -1.11, *SE* = 0.99; EMM _low_ = -2.84, *SE* = 0.99, *p* = 0.005), while no significant difference was found for high intensity images (*p* > 0.05). No significant main effects of Valence or Intensity or other interactions were observed (*p*s > 0.05).

For the P2 component peak, a significant main effect of Intensity was observed [*F* (2, 215) = 5.77, *η2 p* = 0.091, *p* = 0.004]. High intensity images elicited significantly greater P2 amplitude than neutral intensity images (EMM _high_ = 7.47, *SE* = 1.11; EMM _neutral_ = 6.15, *SE* = 1.11, *p* = 0.04), while no significant difference was found for low intensity images (*p* > 0.05). A significant Group × Valence interaction was found [*F* (1, 215) = 4.07, *η2 p* = 0.032, *p* = 0.047]. In the NCID group, negative images elicited greater P2 amplitude than positive images (EMM _positive_ = 0.79, *SE* = 0.94; EMM _negative_ = -1.03, *SE* = 0.94, *p* < 0.001), while no significant difference was observed in the NC group (*p* = 0.28). A significant Valence × Intensity interaction was observed [*F* (2, 215) = 6.40, *η2 p* = 0.032, *p* = 0.003]. Under high intensity conditions, negative images elicited greater P2 amplitude than positive images (EMM _positive_ = -1.59, *SE* = 0.77; EMM _negative_ = -3.43, *SE* = 0.77, *p* = 0.007), while no significant differences were found under neutral or low intensity conditions (*p*s > 0.05). No significant main effects of Valence or Group or other interactions were observed (*p*s > 0.05).

For the LPP component mean, a significant main effect of Valence was observed [*F* (1, 215) = 12.11, *η2 p* = 0.052, *p* = 0.001]. Negative images elicited significantly greater LPP amplitude than positive images (EMM _negative_ = 6.26, *SE* = 0.59; EMM _positive_ = 5.54, *SE* = 0.59, *p* < 0.001). No significant main effects of Group or Intensity or their interactions were found (*p*s > 0.05).

For the EPN component mean, a significant main effect of Valence was observed [*F* (1, 215) = 34.07, *η2 p* = 0.010, *p* < 0.001]. Negative images elicited significantly greater EPN amplitude than positive images (EMM _negative_ = 0.76, *SE* = 0.52; EMM _positive_ = -0.19, *SE* = 0.52, *p* < 0.001). A significant Group × Valence interaction was found [*F* (1, 215) = 10.85, *η2 p* = 0.038, *p* = 0.001]. For positive images, the NC group exhibited greater EPN amplitude than the NCID group (EMM _NC_ = 0.93, *SE* = 0.76; EMM _NCID_ = -1.30, *SE* = 0.71, *p* = 0.037), while no significant difference was observed for negative images (*p* = 0.27). A significant Valence × Intensity interaction was observed [*F* (2, 215) = 9.23, *η2 p* = 0.064, *p* < 0.001]. Under neutral and high intensity conditions, negative images elicited greater EPN amplitude than positive images (neutral intensity: EMM _negative_ = 0.70, *SE* = 0.55 vs. EMM _positive_ = 7.75, *SE* = 0.55, *p* = 0.008; high intensity: EMM _negative_ = 1.38, *SE* = 0.55 vs. EMM _positive_ = -0.50, *SE* = 0.55, *p* < 0.001), while no significant difference was found under low intensity conditions (*p* = 0.51). No significant main effects of Group or Intensity or other interactions were observed (*p*s > 0.05).


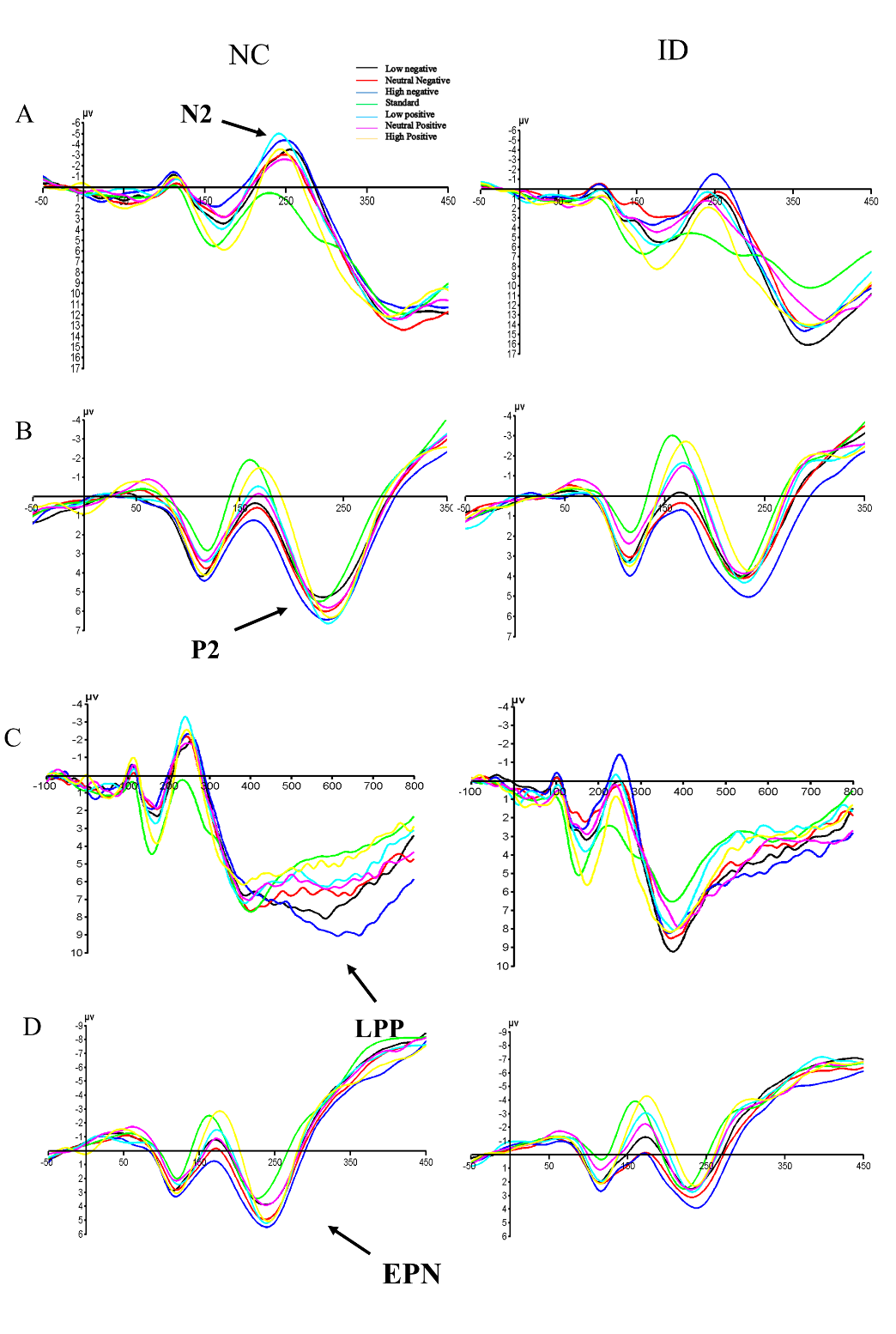


Figure S1. ERP waveforms during emotional processing stages. A, N2 component, waveforms represent the averaged data from F3, F4, Fz, FC1, and FC2 electrode sites. B, P2 component, waveforms represent the averaged data from PO7, PO8, POz, PO5, and PO6 electrode sites. C, LPP component, waveforms represent the averaged data from FC5, FC6, FCz, F7, and F8 electrode sites. D, EPN component, waveforms represent the averaged data from PO7, PO8, P7, P8, O1, and O2 electrode sites.

Supplementary Table S1. Raw data points of phase-amplitude coupling for regional analysis

| Subject ID | Group | ROI | Coupling Strength MI x10^4^ |
| --- | --- | --- | --- |
| 1 | NC | Frontal | 5.285368852 |
| 1 | NC | Central | 6.931576032 |
| 1 | NC | Parietal | 6.781836714 |
| 1 | NC | Temporal | 5.158263523 |
| 1 | NC | Occipital | 8.470631883 |
| 2 | NC | Frontal | 5.246090182 |
| 2 | NC | Central | 4.326075023 |
| 2 | NC | Parietal | 4.616552691 |
| 2 | NC | Temporal | 4.037718499 |
| 2 | NC | Occipital | 4.671723013 |
| 3 | NC | Frontal | 3.755434987 |
| 3 | NC | Central | 4.476146852 |
| 3 | NC | Parietal | 6.279873469 |
| 3 | NC | Temporal | 4.535481215 |
| 3 | NC | Occipital | 5.5245639 |
| 4 | NC | Frontal | 3.801284943 |
| 4 | NC | Central | 5.498753236 |
| 4 | NC | Parietal | 4.897225171 |
| 4 | NC | Temporal | 3.74980985 |
| 4 | NC | Occipital | 5.652432106 |
| 5 | NC | Frontal | 7.482955359 |
| 5 | NC | Central | 5.361924343 |
| 5 | NC | Parietal | 7.618766552 |
| 5 | NC | Temporal | 4.698602485 |
| 5 | NC | Occipital | 6.544947247 |
| 6 | NC | Frontal | 7.329892309 |
| 6 | NC | Central | 7.459188015 |
| 6 | NC | Parietal | 5.272077315 |
| 6 | NC | Temporal | 4.145679073 |
| 6 | NC | Occipital | 4.526509193 |
| 7 | NC | Frontal | 12.25466744 |
| 7 | NC | Central | 13.56109977 |
| 7 | NC | Parietal | 9.091516483 |
| 7 | NC | Temporal | 5.260955903 |
| 7 | NC | Occipital | 3.276739206 |
| 8 | NC | Frontal | 4.560614446 |
| 8 | NC | Central | 5.064385724 |
| 8 | NC | Parietal | 9.268602352 |
| 8 | NC | Temporal | 5.112192977 |
| 8 | NC | Occipital | 4.282704665 |
| 9 | NC | Frontal | 38.61488905 |
| 9 | NC | Central | 33.8000908 |
| 9 | NC | Parietal | 9.866810855 |
| 9 | NC | Temporal | 8.348223782 |
| 9 | NC | Occipital | 5.857795248 |
| 10 | NC | Frontal | 5.685557869 |
| 10 | NC | Central | 5.95896654 |
| 10 | NC | Parietal | 9.632277591 |
| 10 | NC | Temporal | 5.590322578 |
| 10 | NC | Occipital | 11.24399346 |
| 11 | NC | Frontal | 3.441643645 |
| 11 | NC | Central | 3.532196802 |
| 11 | NC | Parietal | 3.954205259 |
| 11 | NC | Temporal | 3.642071191 |
| 11 | NC | Occipital | 3.903429059 |
| 12 | NC | Frontal | 5.487747111 |
| 12 | NC | Central | 4.733199148 |
| 12 | NC | Parietal | 3.551049538 |
| 12 | NC | Temporal | 3.852003249 |
| 12 | NC | Occipital | 4.664625674 |
| 13 | NC | Frontal | 6.544955768 |
| 13 | NC | Central | 9.512183791 |
| 13 | NC | Parietal | 12.24198424 |
| 13 | NC | Temporal | 7.350438318 |
| 13 | NC | Occipital | 16.6218427 |
| 14 | NC | Frontal | 3.854443986 |
| 14 | NC | Central | 4.179345334 |
| 14 | NC | Parietal | 8.292169146 |
| 14 | NC | Temporal | 3.365800037 |
| 14 | NC | Occipital | 3.91951579 |
| 15 | NC | Frontal | 5.421046627 |
| 15 | NC | Central | 7.168905179 |
| 15 | NC | Parietal | 8.177011887 |
| 15 | NC | Temporal | 4.058762374 |
| 15 | NC | Occipital | 8.170751721 |
| 16 | NC | Frontal | 4.883007901 |
| 16 | NC | Central | 3.900949883 |
| 16 | NC | Parietal | 4.020488404 |
| 16 | NC | Temporal | 3.408325103 |
| 16 | NC | Occipital | 2.791359961 |
| 17 | NC | Frontal | 5.211875002 |
| 17 | NC | Central | 6.715992449 |
| 17 | NC | Parietal | 4.880879359 |
| 17 | NC | Temporal | 4.328504969 |
| 17 | NC | Occipital | 4.039878626 |
| 18 | NC | Frontal | 8.580467682 |
| 18 | NC | Central | 8.308709128 |
| 18 | NC | Parietal | 16.95913954 |
| 18 | NC | Temporal | 8.018601226 |
| 18 | NC | Occipital | 35.15844795 |
| 19 | NC | Frontal | 3.963176555 |
| 19 | NC | Central | 4.453099674 |
| 19 | NC | Parietal | 6.530714087 |
| 19 | NC | Temporal | 6.314845739 |
| 19 | NC | Occipital | 4.454557317 |
| 20 | NC | Frontal | 4.437071284 |
| 20 | NC | Central | 4.036789022 |
| 20 | NC | Parietal | 5.952538929 |
| 20 | NC | Temporal | 4.340273205 |
| 20 | NC | Occipital | 4.520513991 |
| 1 | NCID | Frontal | 8.99454042 |
| 1 | NCID | Central | 8.599450187 |
| 1 | NCID | Parietal | 7.034972807 |
| 1 | NCID | Temporal | 5.260049704 |
| 1 | NCID | Occipital | 4.55472452 |
| 2 | NCID | Frontal | 6.105913998 |
| 2 | NCID | Central | 6.099989094 |
| 2 | NCID | Parietal | 7.705314682 |
| 2 | NCID | Temporal | 4.802728449 |
| 2 | NCID | Occipital | 26.50281962 |
| 3 | NCID | Frontal | 9.919371855 |
| 3 | NCID | Central | 15.94767437 |
| 3 | NCID | Parietal | 6.801945107 |
| 3 | NCID | Temporal | 5.641316016 |
| 3 | NCID | Occipital | 4.190336927 |
| 4 | NCID | Frontal | 9.448068095 |
| 4 | NCID | Central | 10.68204158 |
| 4 | NCID | Parietal | 12.33344795 |
| 4 | NCID | Temporal | 7.734778832 |
| 4 | NCID | Occipital | 14.5586632 |
| 5 | NCID | Frontal | 3.561076163 |
| 5 | NCID | Central | 4.069124813 |
| 5 | NCID | Parietal | 4.364760105 |
| 5 | NCID | Temporal | 3.971957984 |
| 5 | NCID | Occipital | 6.69388804 |
| 6 | NCID | Frontal | 10.40406617 |
| 6 | NCID | Central | 7.456834962 |
| 6 | NCID | Parietal | 14.83891725 |
| 6 | NCID | Temporal | 5.064863987 |
| 6 | NCID | Occipital | 36.74585805 |
| 7 | NCID | Frontal | 5.451073477 |
| 7 | NCID | Central | 5.836484726 |
| 7 | NCID | Parietal | 5.004531911 |
| 7 | NCID | Temporal | 4.777186346 |
| 7 | NCID | Occipital | 9.706800592 |
| 8 | NCID | Frontal | 3.091775514 |
| 8 | NCID | Central | 3.154007895 |
| 8 | NCID | Parietal | 3.638482261 |
| 8 | NCID | Temporal | 2.767969412 |
| 8 | NCID | Occipital | 3.430524584 |
| 9 | NCID | Frontal | 3.598109263 |
| 9 | NCID | Central | 3.283657657 |
| 9 | NCID | Parietal | 3.759596819 |
| 9 | NCID | Temporal | 3.826671789 |
| 9 | NCID | Occipital | 3.255731848 |
| 10 | NCID | Frontal | 7.697002972 |
| 10 | NCID | Central | 6.292025961 |
| 10 | NCID | Parietal | 7.218649568 |
| 10 | NCID | Temporal | 7.28602202 |
| 10 | NCID | Occipital | 12.44422138 |
| 11 | NCID | Frontal | 3.494203276 |
| 11 | NCID | Central | 4.098750768 |
| 11 | NCID | Parietal | 5.240199585 |
| 11 | NCID | Temporal | 3.941729064 |
| 11 | NCID | Occipital | 4.659995796 |
| 12 | NCID | Frontal | 3.994388399 |
| 12 | NCID | Central | 4.089949655 |
| 12 | NCID | Parietal | 5.552888817 |
| 12 | NCID | Temporal | 3.663513551 |
| 12 | NCID | Occipital | 3.998462423 |
| 13 | NCID | Frontal | 4.215523917 |
| 13 | NCID | Central | 5.474242581 |
| 13 | NCID | Parietal | 5.84163153 |
| 13 | NCID | Temporal | 4.221653099 |
| 13 | NCID | Occipital | 3.730331565 |
| 14 | NCID | Frontal | 4.347006365 |
| 14 | NCID | Central | 4.682348253 |
| 14 | NCID | Parietal | 5.223526074 |
| 14 | NCID | Temporal | 4.21625403 |
| 14 | NCID | Occipital | 7.657830044 |
| 15 | NCID | Frontal | 3.265360712 |
| 15 | NCID | Central | 3.51789592 |
| 15 | NCID | Parietal | 5.024299066 |
| 15 | NCID | Temporal | 3.194125773 |
| 15 | NCID | Occipital | 5.199255124 |
| 16 | NCID | Frontal | 3.973303644 |
| 16 | NCID | Central | 3.734146505 |
| 16 | NCID | Parietal | 4.253688364 |
| 16 | NCID | Temporal | 3.627916027 |
| 16 | NCID | Occipital | 3.919033106 |
| 17 | NCID | Frontal | 4.824663018 |
| 17 | NCID | Central | 4.391397707 |
| 17 | NCID | Parietal | 5.133490586 |
| 17 | NCID | Temporal | 3.943607006 |
| 17 | NCID | Occipital | 7.648523408 |
| 18 | NCID | Frontal | 4.535389908 |
| 18 | NCID | Central | 6.167519098 |
| 18 | NCID | Parietal | 8.292270552 |
| 18 | NCID | Temporal | 5.015523944 |
| 18 | NCID | Occipital | 6.925394818 |
| 19 | NCID | Frontal | 4.429100173 |
| 19 | NCID | Central | 5.880032493 |
| 19 | NCID | Parietal | 8.594606924 |
| 19 | NCID | Temporal | 4.959757732 |
| 19 | NCID | Occipital | 6.28520109 |
| 20 | NCID | Frontal | 4.164619203 |
| 20 | NCID | Central | 4.677357054 |
| 20 | NCID | Parietal | 13.40533932 |
| 20 | NCID | Temporal | 3.790932017 |
| 20 | NCID | Occipital | 13.95115077 |
| 21 | NCID | Frontal | 12.35075289 |
| 21 | NCID | Central | 9.395041024 |
| 21 | NCID | Parietal | 7.733577844 |
| 21 | NCID | Temporal | 5.064127716 |
| 21 | NCID | Occipital | 6.235687556 |
| 22 | NCID | Frontal | 5.691543653 |
| 22 | NCID | Central | 3.571462494 |
| 22 | NCID | Parietal | 4.395031724 |
| 22 | NCID | Temporal | 3.84899332 |
| 22 | NCID | Occipital | 5.132682661 |
| 23 | NCID | Frontal | 6.276840956 |
| 23 | NCID | Central | 8.431327884 |
| 23 | NCID | Parietal | 8.659508971 |
| 23 | NCID | Temporal | 4.732500537 |
| 23 | NCID | Occipital | 11.44862909 |
